# Perceptions of normal and abnormal ejaculatory latency times: an observational study in Ghanaian males and females

**DOI:** 10.1186/s40001-015-0169-6

**Published:** 2015-09-04

**Authors:** Nafiu Amidu, William K. B. A. Owiredu, Peter P. M. Dapare, Benedict B. Antuamwine

**Affiliations:** Department of Biomedical Laboratory Sciences, School of Allied Health Sciences, University for Development Studies, Tamale, Ghana; Department of Molecular Medicine, School of Medical Sciences, College of Health Sciences, Kwame Nkrumah University of Science and Technology, Kumasi, Ghana

**Keywords:** Intravaginal ejaculatory latency, Perception, Premature ejaculation, Ghana

## Abstract

**Background:**

This cross-sectional study aimed at quantifying the perceptions of Ghanaian men and women on how long they thought sex should last, from intromission until ejaculation.

**Method:**

A random sample of 568 heterosexual men and women within the Kumasi Metropolis was surveyed from December 2009 to February 2010. The question of primary interest in the present study includes perceived intravaginal ejaculatory latency (IELT), in minutes, for four different conditions: “adequate,” “desirable,” “too short,” and “too long” IELT.

**Results:**

The interquartile range for the respondent’s judgment of an “adequate” length for IELT was from 7.0 to 20.3 min; “desirable” from 10.0 to 25.0 min; “too short” from 2.0 to 5.0 min; “too long” from 10.5 to 60.0 min. However, the “actual” IELT (i.e. what the respondents are capable of doing) as found in this study was from 6 to 15 min. Ghanaian perceptions about ejaculatory latencies were in part consistent with data from Germany and contrary to data from the USA on ejaculatory latency and were not affected by age or educational level.

**Conclusion:**

These results suggest that the average Ghanaian believes that intercourse that lasts 7.0–25.0 min is normal. Dissemination of the present finding to the public may modify their expectations for IELT which will lead to a realistic replica of sexuality and hence help prevent sexual disappointments and dysfunctions. It will also be beneficial to couples who are being treated for sexual problems by normalizing their expectations.

## Background

An evidence-based definition of premature ejaculation is important for the diagnosis and treatment of men who complain of premature ejaculation (PE) [1]. The consecutive Work Groups on Sexual Disorders of the Diagnostic and Statistical Manual of Mental Disorders (DSM) gained popularity for its classification of PE. These definitions by DSM, however, have been heavily criticized in recent studies for its vagueness and absence of a short ejaculation time criterion cutoff score [2]. The International Society for Sexual Medicine by consensus formulated the first evidence-based definition of lifelong PE in 2008, where short ejaculation time was defined as an ejaculation occurring within 1 min after vaginal penetration, with an inability to delay ejaculation and with personal negative consequences such as bother or avoidance of sexual activities [3].

A lot of men, however, complain of PE at longer durations of the intravaginal ejaculation latency time (IELT). To include these men in a classification system of PE, Waldinger et al. [4, 5] proposed the existence of two other PE subtypes; natural variable PE and premature-like ejaculatory dysfunction, in addition to the earlier proposed subtypes, lifelong and acquired PE. In contrast to men with lifelong PE, men with premature-like ejaculatory dysfunction complain of PE while having normal or even long durations of IELT. Notably, Waldinger emphasized that the four PE subtypes were characterized by different etiology and pathophysiology [4].

In two stopwatch-measured surveys in the general male population of five Western countries, the median IELT appeared to be 5.4 and 6.0 min, respectively [5, 6]. In these surveys, the prevalence of IELTs of less than 1 min was about 2.5 %, whereas 28 % of the investigated men considered themselves to have a too short IELT. The mean IELT of these men was 4.9 min. The existence of the four PE subtypes has been confirmed by Serefoglu et al. [7, 8] who showed that the prevalence of lifelong PE is low in the general Turkish male population and high in an outpatient clinic for urology. In addition, and as predicted by Waldinger, the prevalence of premature-like ejaculatory dysfunction is relatively high in the general Turkish male population and low in an outpatient clinic for urology [7, 8].

Despite the majority of studies on the duration of IELTs with or without stopwatches occurring within Western and Asian countries, there are no data so far derived from an African perspective. Due to the variations existing between Western and African countries in terms of cultural differences, socioeconomic levels and the quality of psychosexual relationships, which may influence the perception of how long IELT should last during intercourse, it will be interesting to find out how this will be perceived in one or more African countries.

Additionally, it will be fascinating to know the general perception of men and women regarding normal intercourse duration, one that may be a point of contention among both sexes. The aim of the current study among a sample of Ghanaian men and women was therefore to investigate the Ghanaian perception on normal and abnormal intercourse duration. To our knowledge, this is the first study on the perception of IELT conducted among an African population.

## Methods

### Study population

This epidemiological cross-sectional study population comprised heterosexual, adult Ghanaian male and female subjects of age 17–64 years. Questionnaires were distributed to a total of 900 heterosexual men and women randomly within the Kumasi Metropolis who had attained at least basic education (i.e. at least junior high school). Randomization of the study population was carried out by handing out questionnaires to target groups at schools, canteens, bus terminals, automechanic shops and offices. Kumasi is the second largest city in Ghana after the capital (Accra) with a population of about 1.5 million. The inhabitants are predominantly Christians, forming about 80 % of the population and have Ashanti as the largest ethnic group. The participation of the respondents who were all indigenes of Ghana was voluntary and informed consent was obtained from each of them. The study was approved by the local Committee on Human Research, Publication and Ethics (CHRPE).

### Measurements

Questions regarding perception of normal and abnormal IELT were adopted and modified from a study among sex therapists conducted in the USA and Canada [9]. The respondents were asked to provide information on their demographics (age, sex, occupation, educational level, marital status, etc.) and were to give their opinion on questions about IELT with responses such as “too short,” “adequate,” “desirable,” or “too long.”

Additionally, the respondents were asked to give their opinion on several other questions including; “What is an *adequate* amount of time to elapse in sex from penile penetration of the vagina to ejaculation?” which was asked in four different ways, with the italicized word changing from *adequate*, to *desirable*, to *too short* and then to *too long*. Respondents were also asked to estimate the time in minutes that they normally stay in sex from penile penetration of the vagina to ejaculation, the IELT [10]. This was an estimated time response.

### Statistical analysis

The data were presented as mean ± SD or percentages. Continuous data were analyzed using unpaired *t* test, while categorical variable were analyzed using Fischer’s exact test. In all statistical tests, a value of *P* < 0.05 was considered significant. All analysis were performed using SigmaPlot for Windows, Version 11.0, (Systat Software, Inc. Germany) http://www.systat.com.

## Results

Six hundred and twenty two (69 %) of the 900 distributed questionnaires were returned. Twenty-three (3.7 %) of these respondents indicated no sexual experience and felt unable to provide responses to the questions. Thirty-one respondents (5 %) did not provide a time estimate response for at least one of the IELT questions. However, they indicated that the interpretation of IELT depends on the partner and/or the number of times the sex act was performed. The remaining 568 (91.3 %) respondents had a mean age ± SD of 28.7 ± 0.6 years (range 17–60 years) and were fairly distributed between married (~43 %) and never married (single) subjects. Majority of the respondents were males (85 %) and had attained tertiary education (86 %) while very few of the respondents smoked (7.4 %) as shown in Table 1.

**Table 1:** Demographic characteristics of the studied population stratified by gender

Variable	Total (568)	Male (483)	Female (85)	*P* value
Age (years)	28.7 ± 0.6	29.7 ± 0.7	22.8 ± 0.8	<0.0001
Married	242 (42.6 %)	217 (44.9 %)	25 (29.4 %)	0.0009
Educational level				
Basic	7 (1.2 %)	7 (1.5 %)	0 (0.0 %)	0.2641
Secondary	97 (17.1 %)	74 (15.3 %)	23 (27.1 %)	0.008
Tertiary	464 (81.7 %)	402 (83.2 %)	62 (73.0 %)	0.0237
Exercise				
Yes	291 (51.2 %)	252 (52.2 %)	39 (45.9 %)	0.2089
Smoking				
Yes	42 (7.4 %)	42 (8.7 %)	0 (0.0 %)	0.0047
Alcohol intake				
Yes	321 (56.5 %)	294 (60.9 %)	27 (31.8 %)	<0.0001

Results are presented as mean ± standard deviation

*IELT* estimated intravaginal ejaculatory latency time

When a respondent’s response is in a range (e.g. 3–7 min), the midpoint of the range (e.g. 5 min) is calculated and used as the response. Though the female respondents were significantly younger than their male counterparts and the married respondents were also significantly older than the single respondents, their perception about IELTs were generally similar. However, the perception of married respondents about “adequate” IELT was significantly higher (17.9 ± 1.9) than those who were single (13.7 ± 1.0) (*P* = 0.0404) as shown in Table 2.

**Table 2:** General characteristic of the studied population stratified by gender and marital status

Parameters	Total	Male	Female	*P* value	Single	Married	*P* value
Age (years)	28.7 ± 0.6	29.7 ± 0.7	22.8 ± 0.8	<0.0001	25.2 ± 0.6	33.1 ± 1.0	<0.0001
Actual IELT (min)	9.4 ± 1.6	9.6 ± 1.8	8.2 ± 2.3	0.7653	10.5 ± 2.7	8.1 ± 1.4	0.4490
Adequate IELT (min)	15.4 ± 1.0	15.1 ± 1.1	17.1 ± 2.8	0.4796	13.7 ± 1.0	17.9 ± 2.0	0.0404
Desirable IELT (min)	18.8 ± 1.3	18.8 ± 1.4	19.1 ± 3.3	0.9383	18.2 ± 1.5	19.6 ± 2.3	0.5792
Too short IELT (min)	4.9 ± 0.6	4.8 ± 0.6	5.4 ± 1.5	0.6896	4.3 ± 0.5	5.6 ± 1.1	0.2404
Too long IELT (min)	36.7 ± 2.4	36.4 ± 2.8	38.4 ± 4.2	0.7769	36.0 ± 3.5	37.7 ± 3.3	0.7295

Results are presented as mean ± standard deviation

*IELT* estimated intravaginal ejaculatory latency time

The questions of primary interest in the present study involved respondents’ definitions of “adequate” and “desirable” IELTs. The mean ± SD for these variables were, respectively, 15.4 ± 1.0 and 18.8 ± 1.3 min, with interquartile ranges (IQRs) 7.0–20.3 and 10.0–25.0 min, respectively. (The IQR represents the responses of the middle 50 % of respondents, the range from the 25th percentile to the 75th percentile of responses.) The respondents were also asked the definitions for IELTs that were “too short” or “too long”. The mean ± SD times for these were 4.9 ± 0.6 and 36.7 ± 2.4 min, respectively. IQRs for these variables were 2.0 to 5.0 and 10.5 to 60.0 min, respectively. The mean as well as the IQR for the “actual” IELT (i.e. what the respondents are capable of doing) as found in this study were 9.4 ± 1.6 and 6 to 15 min, respectively (Table 2).

**Fig. 1:**
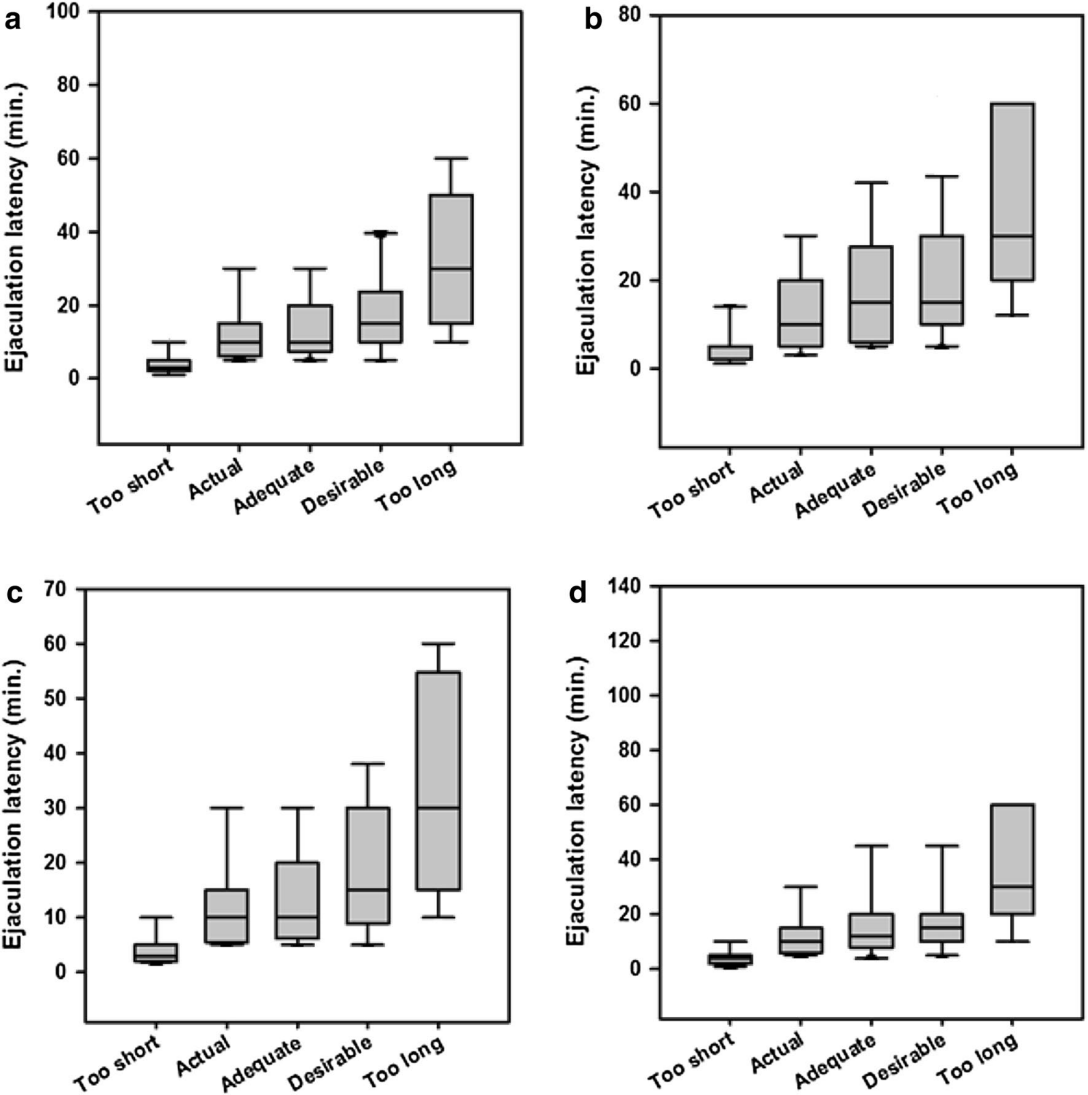
Box plots of respondent’s ratings of lengths of different categories of ejaculatory latencies. The *lower* and *upper* margins of the *box* represent the 25th and 75th percentiles, with the extended arms representing the 10th and 90th percentiles, respectively. The median is shown as the *horizontal line* within the *box*. *A* male, *B* female, *C* single and *D* married

Box plots showing the medians, IQRs and range of scores for all latency questions based on gender and marital status are shown in Fig. 1.

Overall, only 25 % of men and women perceived “adequate” IELT to last for 3–7 min, while about 74 % perceived “adequate” IELT to last for more than 3–7 min (Table 2).

Though about 68 % of single subjects perceived “adequate” sex to last for more than 3–7 min, about 50 % perceived “desirable” sex to last for more than 13 min, 57 % perceived “too short” sex to be greater than 2 min and 38 % perceived “too long” sex to last for more than 30 min. The figures were 81 %, 66 %, 71 % and 45 %, respectively for “adequate”, “desirable”, “too short” and “too long” among married respondents (Table 3).

**Table 3:** The proportions of respondents who perceived intravaginal ejaculation latency above (i.e. high) and below (i.e. low) the reference interval for “adequate”, “desirable”, “too short” and “too long” IELT

Parameters	Total	Male	Female	*P* value	Single	Married	*P* value
Adequate (3–7)							
Low	4/556 (0.7 %)	4/472 (0.8 %)	0/84 (0.0 %)	1.0000	0/324 (0.0 %)	4/236 (1.7 %)	0.0311
High	412/556 (74.1 %)	352/472 (74.6 %)	60/84 (71.4 %)	0.5889	220/324 (67.9 %)	192/236 (81.4 %)	0.0005
Desirable (7–13)							
Low	92/568 (16.2 %)	40/488 (16.4 %)	12/84 (14.3 %)	0.0970	48/324 (14.8 %)	40/236 (16.9 %)	0.5568
High	316/568 (55.6 %)	272/488 (55.7 %)	44/84 (52.4 %)	0.6349	160/324 (49.4 %)	156/236 (66.1 %)	<0.0001
Too short (1–2)							
Low	0/556 (0.0 %)	0/476 (0.0 %)	0/84 (0.0 %)	1.0000	0/324 (0.0 %)	0/236 (0.0 %)	1.0000
High	352/556 (63.3 %)	302/476 (63.4 %)	50/84 (59.5 %)	0.5406	184/324 (56.8 %)	168/236 (71.2 %)	0.0005
Too long (10–30)							
Low	32/564 (5.7 %)	28/480 (5.8 %)	4/84 (4.8 %)	1.0000	20/324 (6.2 %)	12/240 (5.0 %)	0.5863
High	232/564 (41.1 %)	192/480 (40.0 %)	40/84 (47.6 %)	0.2293	124/324 (38.3 %)	108/240 (45.0 %)	0.0826

To investigate whether demographic variables (age, marital status and educational level) were associated with perceptions of IELTs (too short, adequate, desirable or too long), the Pearson correlation matrix was employed. Apart from age that had a significant but negative correlation (*r* = −0.23; *P* < 0.01) with education, none of the demographic variables showed a statistically significant association with any of the latency variables. The latency variables, however, generally gave a significant positive correlation with each other as shown in Table 4.

**Table 4:** Pearson correlation coefficients of demographic variables and perceptions of ejaculatory latencies

Marital status		Education	Actual	Adequate	Desirable	Too short	Too long
Age	0.06	−0.23**	0.05	−0.05	−0.02	−0.07	−0.03
Marital status		0.07	0.06	−0.15	−0.11	−0.09	−0.05
Education			0.04	0.07	−0.12	0.08	0.05
Actual				0.16	0.35***	0.24**	0.56***
Adequate					0.56***	0.21*	0.42***
Desirable						0.23**	0.68***
Too short							0.25**

* Correlation is significant at the 0.05 level (2-tailed)

** Correlation is significant at the 0.01 level (2-tailed)

*** Correlation is significant at the 0.001 level (2-tailed)

## Discussion

The present study which is the first of its kind in our community, a very conservative community, compared the actual, adequate, desired, too short and too long IELT of heterosexual men and women who were either single or married. Even though estimates of the duration of IELT experienced by men and women do not differ significantly from their perceived duration of adequate, desired, too short and too long IELT, the men’s estimates of actual IELT they experience was about 1.4 min longer than that of women. Though the difference did not reach a significant level, the women consistently estimated higher perceived IELTs than their male counterparts (Table 2), a trend that was similarly observed by Waldinger and associates among a Dutch cohort of men with lifelong PE [11].Whereas this finding is similar to data from Germany, it is at variance with data from the USA [12]. This could partly be due to the fact that women are more open about their sexual life than men, at least in relationships and dating [13, 14]. Cultural as well as methodological differences could also contribute to this disparity.

Our study shows that the IQR for perceived adequate IELT is around 7.0–20.3 min. This is longer than what Western sex therapists deem as adequate IELT and borders around what many experienced sex therapists may consider to be too long [9]. The IQR of IELTs perceived by experienced Western sex therapists to be “adequate” ranges from 3 to 7 min.

There is also an overlap between what respondents consider to be an adequate latency and a “desirable” IELT, with the respondents perceiving longer IELTs (IQR = 10.0–25.0 min) as more desirable. Combining these two IQRs, it sounds reasonable to say that an IELT of 7–25 min is perceived by respondents as normal. This may have consequences for men who are not satisfied with their ejaculation time and may erroneously be classified as having premature ejaculation when their actual ejaculation time is less than their desirable IELT. The current study underlines the existence of the PE subtype, premature-like ejaculatory dysfunction as recently proposed by Waldinger et al. [11], where both men and women in the general population who have normal or even long IELT duration are not satisfied with IELT values which are normal in the general male population as has been measured by stopwatch in two population surveys [5, 6]. Our findings can also be compared to previous reports of the same authors [5, 6] that about 30 % of men in Western countries are not satisfied with normal IELT values.

In the current group of African subjects, married respondents do not only perceive longer time as being “adequate,” “desirable,” “too short,” or “too long” IELTs, but also a significantly higher proportion (about 13, 17, 14 and 7 % more than the single respondents, respectively) have a perceived time which is more than what Western sex therapists recommend [9]. The reasons for these discrepancies are not clearly shown from this study, but may be culturally influenced. Future research with objective methods, such as the use of a stopwatch, will show that the actual IELT duration in certain African cohorts is indeed longer than that generally measured in Western countries and these differences may perhaps be explained by biological or genetic factors [15]. However, in general, the actual ejaculation latency time depends on a variety of contextual, psychological, behavioral and relationship partner variables [16].

On comparing actual to desired time, both sexes wanted an increase in the length of both variables. The men desired an increase from a reported time of 9.6 min in intercourse to a desired length of 18.8 min, whereas the women desired an increase from 8.2 to 19.1 min. With both men and women desiring that intercourse lasts about twice as long as the self-reported length, it is conceivable that this may lead to distress, displeasure and ultimately to the purchase of sex-enhancing medication as observed currently among Ghanaians (Amidu, personal observation). However, one should be cautious not to diagnose these men as having PE. Recently, men who are not satisfied with their IELT while having a normal or even long IELT duration have been classified as having premature-like ejaculatory dysfunction [1]. According to Waldinger et al, this PE subtype has a clearly different etiology and pathogenesis than lifelong PE or acquired PE [4]. This Ghanaian men cohort is most likely the group with premature-like ejaculatory dysfunction. In the general male population, the prevalence of this PE subtype is high, whereas in clinics of sexual medicine, it is rather low. The prevalence of sexual dysfunction (SD) and PE among Ghanaian men are 66 and 65 % respectively [17], 73 % SD rate among the women [18] and 80 % PE rate among men presenting with various medical conditions [19].

From this study, respondent characteristics did not influence their perceptions of normal and abnormal IELTs. Neither age nor marital status was associated with perceived IELT, suggesting that the respondents’ judgments were not based on their personal experiences or marital status. Similarly, perceived latencies did not vary with educational level. This suggests that for these respondents, at least continuing education gave them a shared perspective.

For people with existing SD, it will be advisable for their sex therapists in Ghana to assess the actual latency, since self-estimate may be discrepant from actual latency. Afterward, the patient’s desired latency should be determined and this objective discussed in the context of the data reported here and that of the sex therapists (i.e. sex therapists consider coitus that lasts as little as 3 min to be of adequate length) [9]. This can help shift the patient away from experiencing distress or interpersonal difficulty and prevent the patient from perceiving his or her situation as a problem [20].

The study is in agreement with that of Waldinger [20] who suggested that various counseling methods, and not drug treatment, should be used in treating individuals who present with premature-like ejaculatory disorders due to their actual IELTs being perceived as rapid ejaculation. Even though a person’s understanding of sexual functioning is related to inherent standards and beliefs, it can greatly be modified by the type of formal and informal education received from the society [9], including pornographic movies. Since expectation is known to be determined by stereotypes and not reality [21], dissemination of the results of the present study can help modify individual expectations about sexual functioning and performance. By dissociating expectations from fantasy to a realistic replica of sexuality, the present results may help prevent sexual disappointments and dysfunctions.

One major limitation of this study is that participants were asked to reduce a complex human sexual behavior to a single number. Despite the fact that the majority of respondents provided the requested values, such values would not be able to explain the complexity of human behavior. Due to the difficulty in determining at what time during intercourse the reading starts as the couple may move to and from vaginal encapsulation of the penis [21], the usage of intravaginal ejaculatory latency as an outcome measure is further confounded. Furthermore, this study used self-report to measure time and it is possible that there may be a biased estimate of the length of intercourse.

## Conclusion

The results from the present study provides a realistic as well as a baseline model of Ghanaian sexuality and would be useful both in treating people with sexual concerns and dysfunctions, and with wider applications in preventing the onset of SD.
